# 297. Evaluation of Oral Versus Intravenous Beta Lactams for the Treatment of Osteomyelitis

**DOI:** 10.1093/ofid/ofaf695.099

**Published:** 2026-01-11

**Authors:** Mikayla Jovanovich, Linda Yang, Teri Hopkins, Paola Carcamo, Christopher R Frei, Elizabeth Walter, Jose Cadena

**Affiliations:** South Texas Veterans Healthcare System, San Antonio, Texas; VA North Texas Health Care System, Dallas, Texas; South Texas Veterans Healthcare System, San Antonio TX;UT Long School of Medicine, San Antonio TX; UT Austin College of Pharmacy, Austin TX, San Antonio, Texas; South Texas Veterans Healthcare System, San Antonio, Texas; University of Texas, San Antonio, Texas; Audie L. Murphy Memorial Veterans Hospital, San Antonio, Texas; South Texas Veterans Health Care System, UT Health San Antonio, San Antonio, Texas

## Abstract

**Background:**

Limited data exist regarding the use of oral beta-lactams for the treatment of osteomyelitis (OM). This study evaluated the effectiveness of oral versus intravenous (IV) beta-lactams for the treatment of OM.

**Figure:**
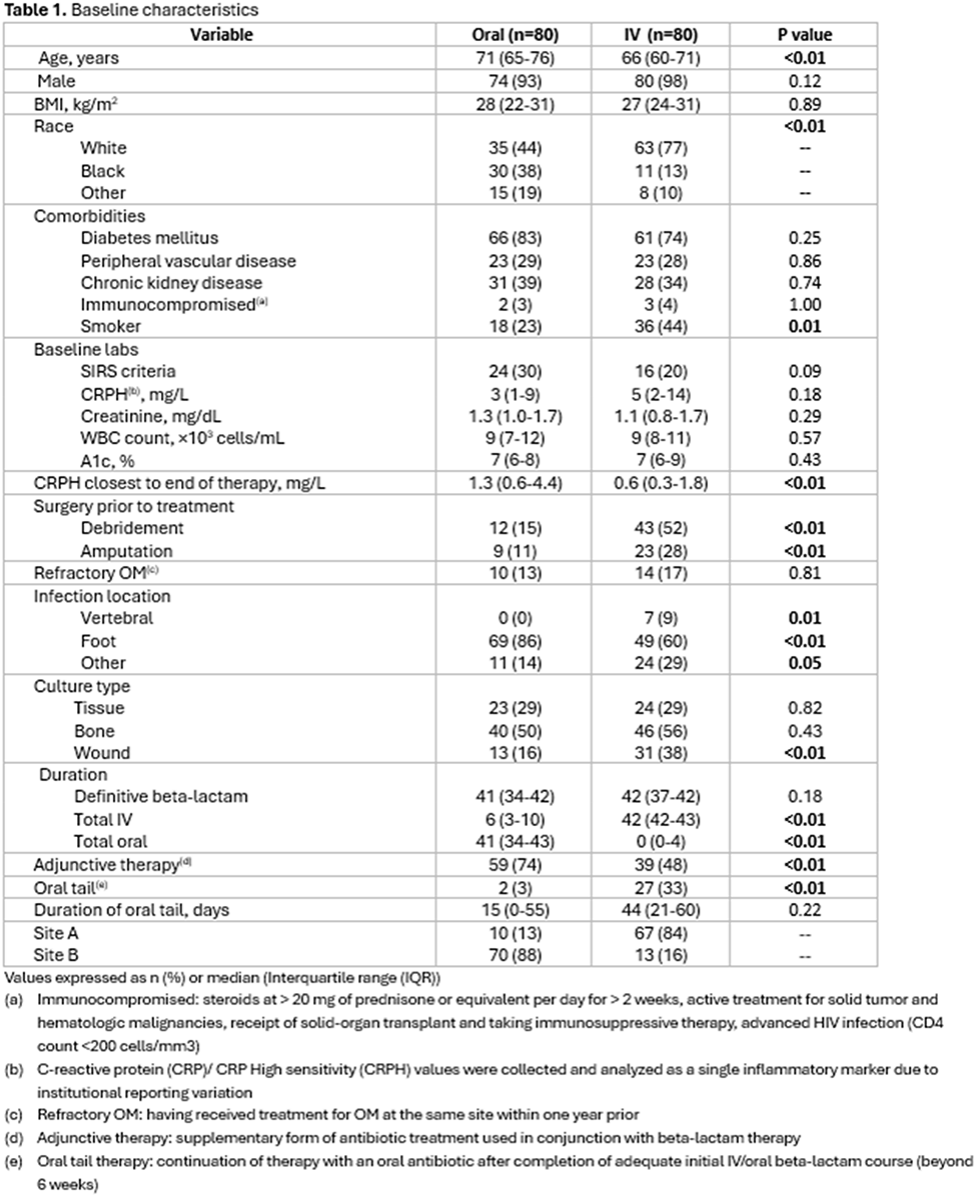

**Methods:**

We conducted a multicenter, retrospective, cohort study of Veterans Affairs (VA) patients who received either oral or IV beta-lactams for the treatment of OM between January 1, 2017 and December 31, 2023. The primary outcome was treatment failure within one year from first dose of definitive therapy, defined as the need to restart or change antimicrobial therapy, additional surgical intervention at the same site, readmission for OM at the same site, or all-cause mortality. Secondary outcomes included hospital length of stay, significant adverse drug events, and frequency of line complications in patients receiving IV therapy. Patients were matched based on BMI > 30 kg/m^2^, A1c > 8%, and baseline surgical amputation. Chi-square, Fischer’s Exact, and Wilcoxon Rank Sum tests were used to compare outcomes. Multivariate logistic regression models assessed associations between treatment route and outcomes, adjusting for significantly different baseline characteristics.

**Results:**

Data from 160 patients were included for analysis (80 in each group). Table 1 depicts baseline characteristics. No statistically significant difference was observed in treatment failure between oral and IV beta-lactams (50% vs 45%; p=0.53). All-cause mortality was higher in the oral group (26% vs 6%; < 0.01), while IV-treated patients required more therapy modifications (6% vs 20%; p < 0.01), additional surgeries (15% vs 30%; p=0.03), and readmissions related to OM (14% vs 38%; p < 0.01). Route was not independently predictive of treatment failure (0.53) or overall mortality (p=0.27) in multivariate logistic regression models. No significant differences were observed in secondary outcomes, including hospital length of stay, significant adverse drug events, or line-related complications.

**Conclusion:**

Oral beta-lactams may be a viable OM treatment option. Higher mortality observed in the oral group likely reflected more patients in hospice or preferring less aggressive care. Future analyses will refine the matching methodology to further balance baseline characteristics.

**Disclosures:**

All Authors: No reported disclosures

